# Supplementary material for: Unveiling the Mechanism of Action of Palmitic Acid, a Human Topoisomerase 1B Inhibitor from the Antarctic Sponge Artemisina plumosa
Source: Int J Mol Sci. 2025 Feb 26;26(5):2018. doi: 10.3390/ijms26052018 (PMC11900379; doi:10.3390/ijms26052018)
Supplement: Supplementary file 1 [file ijms-26-02018-s001.zip › ijms-3470884-supplementary.pdf]

SUPPLEMENTARY MATERIALS

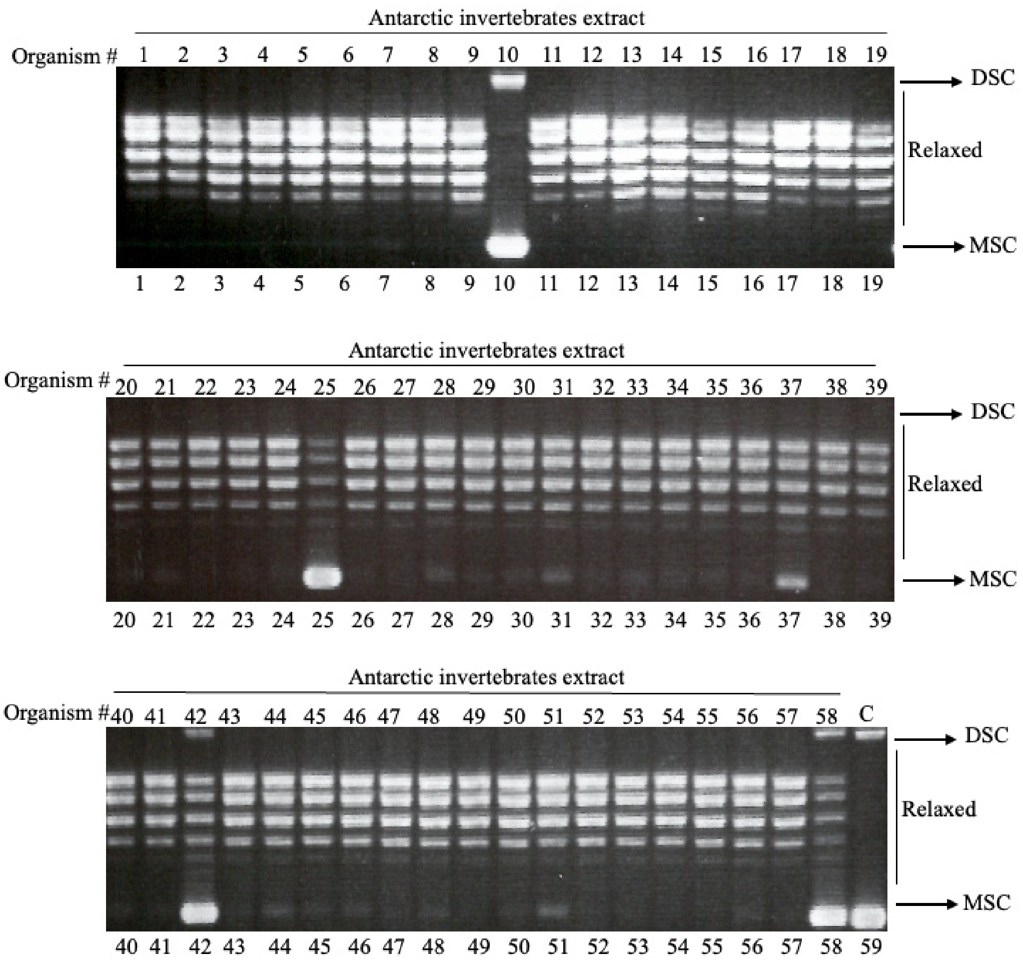

Supplementary Figure 1. Antarctic organisms crude extract effect on DNA relaxation assay. Relaxation of supercoiled DNA in presence of hTOP1 and different extracts from Antarctic organism (lanes 1-58). The reaction products are resolved on agarose gel and visualized with EtBr. DSC dimer supercoiled DNA plasmid; MSC monomer super-coiled DNA plasmid and C negative control.

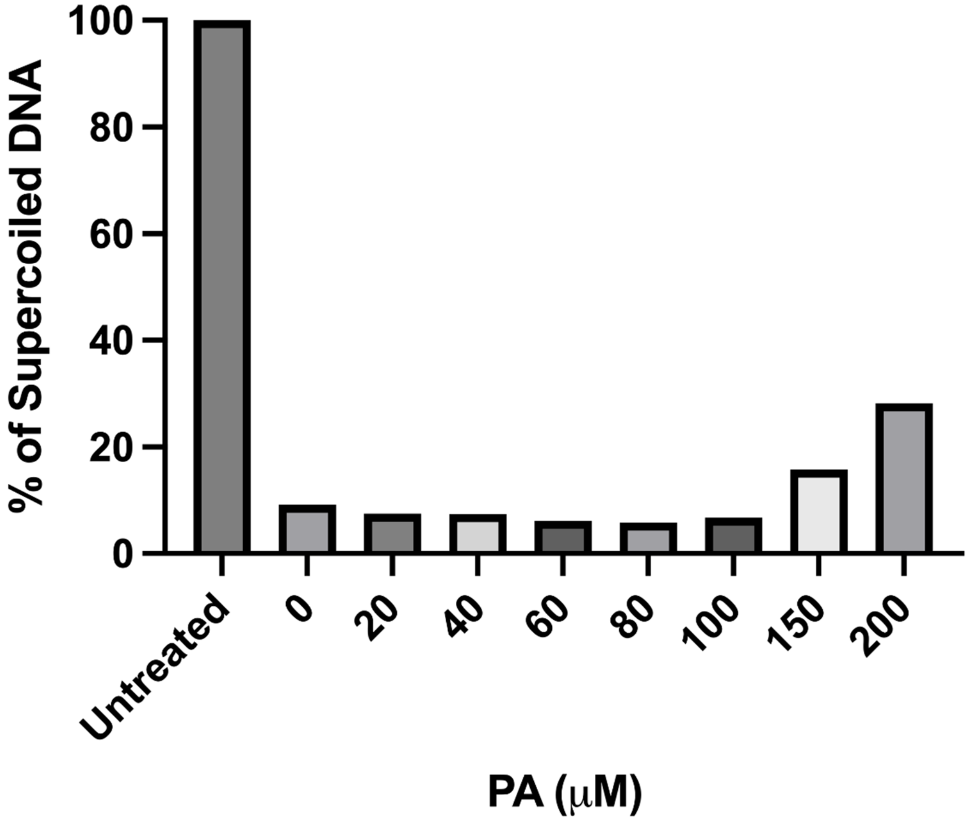

Supplementary Figure 2. Quantification of supercoiled DNA in the presence of palmitic acid. The graph shows the percentage of supercoiled DNA as a function of palmitic acid concentration. Untreated represents supercoiled DNA in the presence of PA and in the absence of hTOP1.

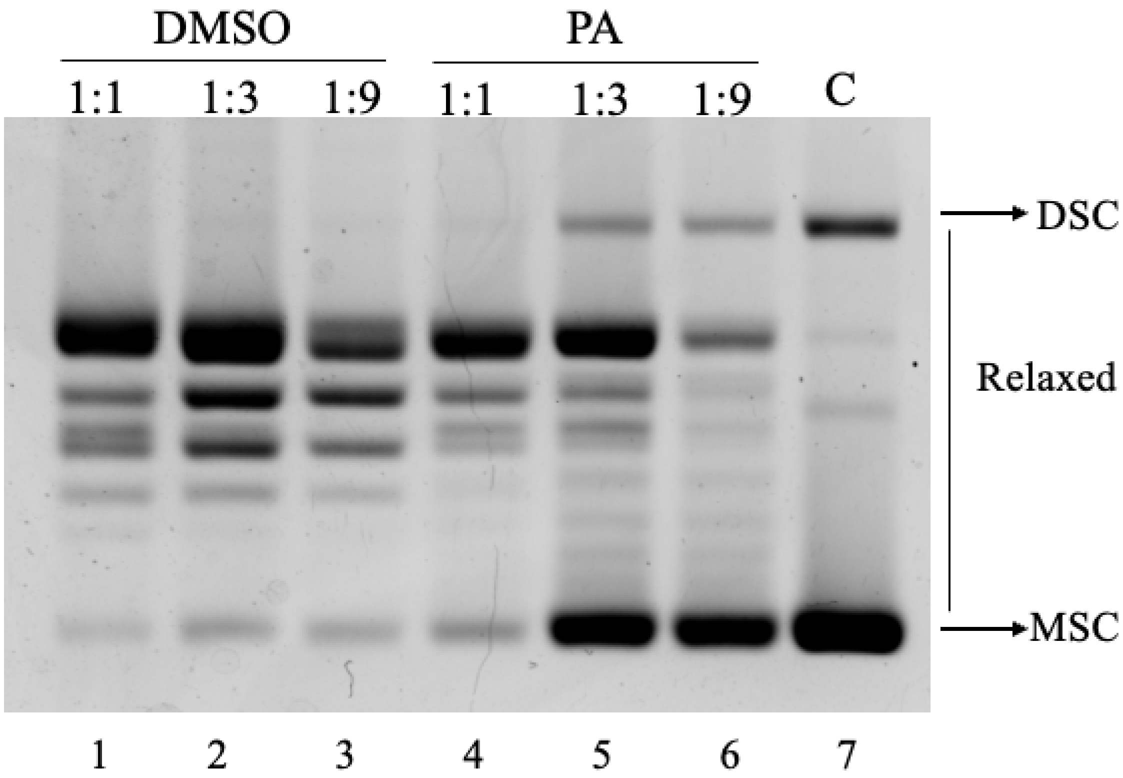

Supplementary Figure 3. Evaluation of the irreversibility of PA mechanism of action**.** Relaxation assay of hTOP1 preincubated with DMSO (lanes 1-3) or 100 μM PA (lanes 4-6) for 15 min and then diluted 3- or 9-fold. Lane 7, no protein added. The reaction products were resolved on agarose gel and visualized with ethidium bromide. DSC dimer supercoiled DNA plasmid; MSC monomer super-coiled DNA plasmid and C negative control.

A

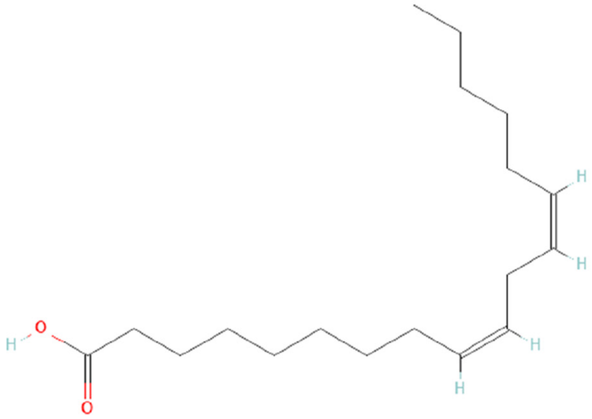

B

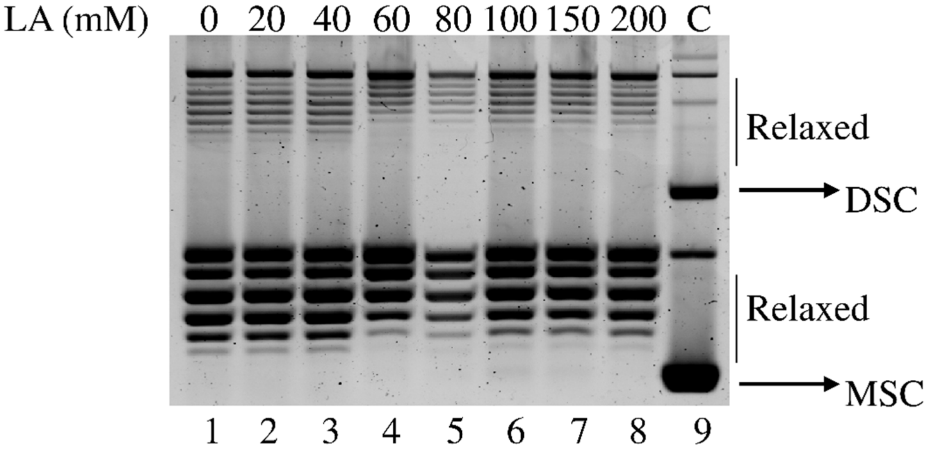

Supplementary Figure 4. LA effect on DNA relaxation assay. (A) 2D structure of LA. (B) Relaxation of negative supercoiled DNA plasmid by hTOP1 at increasing LA concentrations (lanes 2–8), lane 1, DMSO and lane 9, negative control. The reaction products are resolved on agarose gel and visualized with ethidium bromide. DSC dimer supercoiled DNA plasmid; MSC monomer super-coiled DNA plasmid and C negative control.

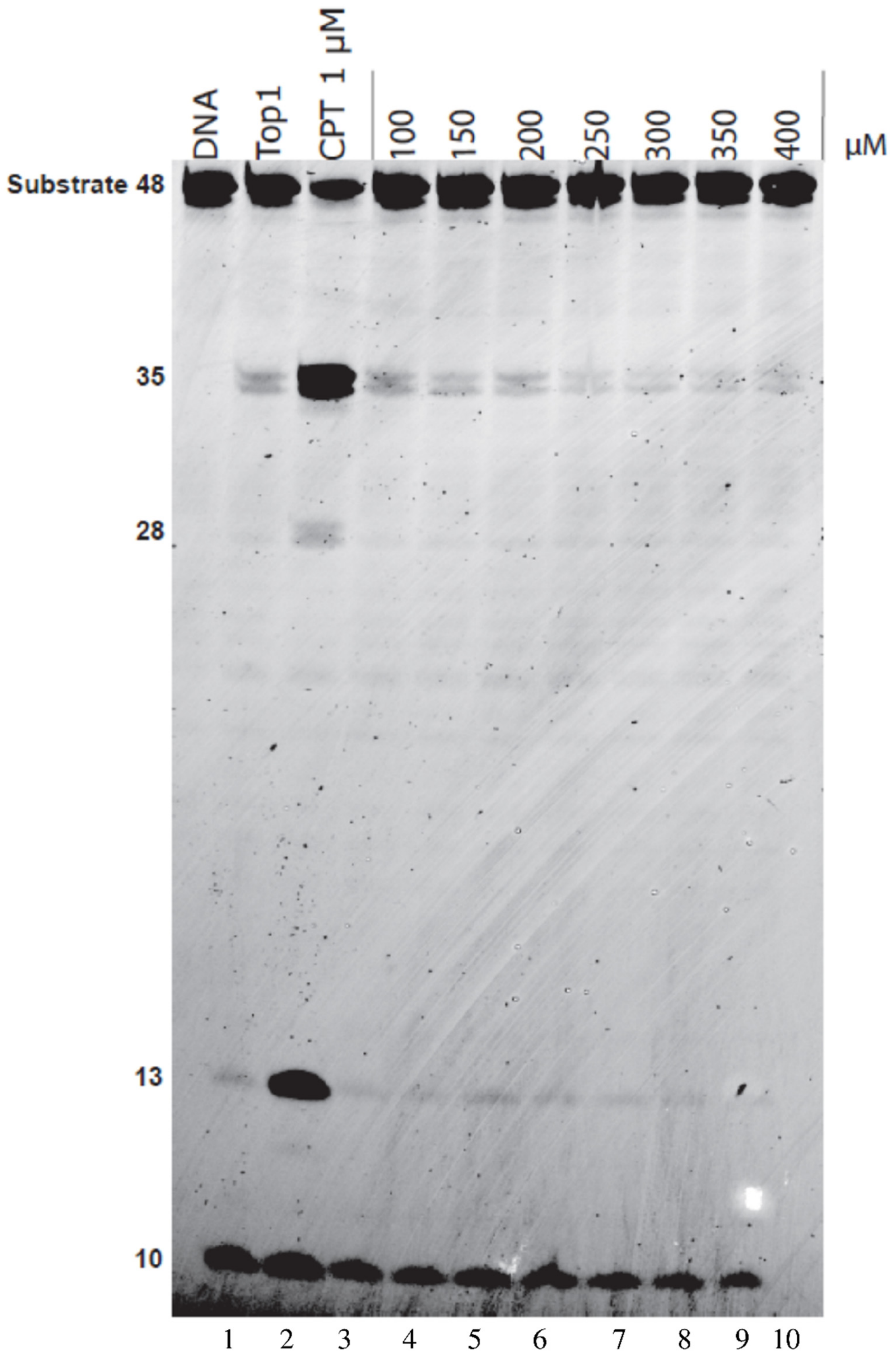

Supplementary Figure 5. Dose dependent hTOP1–DNA cleavage complex reversal assay. A polyacrylamide gel showing the kinetics of formation of PA and CPT-induced hTOP1-mediated DNA cleavage complexes. A 3'-6-FAM end-labeled 48 bp oligonucleotide was incubated with hTOP1 at 25°C for 20 minutes, either with or without 1 μM CPT and 100 μM, 150 μM, 200 μM, 250 μM, 300 μM, 350 μM, 400 μM of PA. The reversal of DNA cleavage was achieved by adding 0.35 M NaCl and was monitored over time.

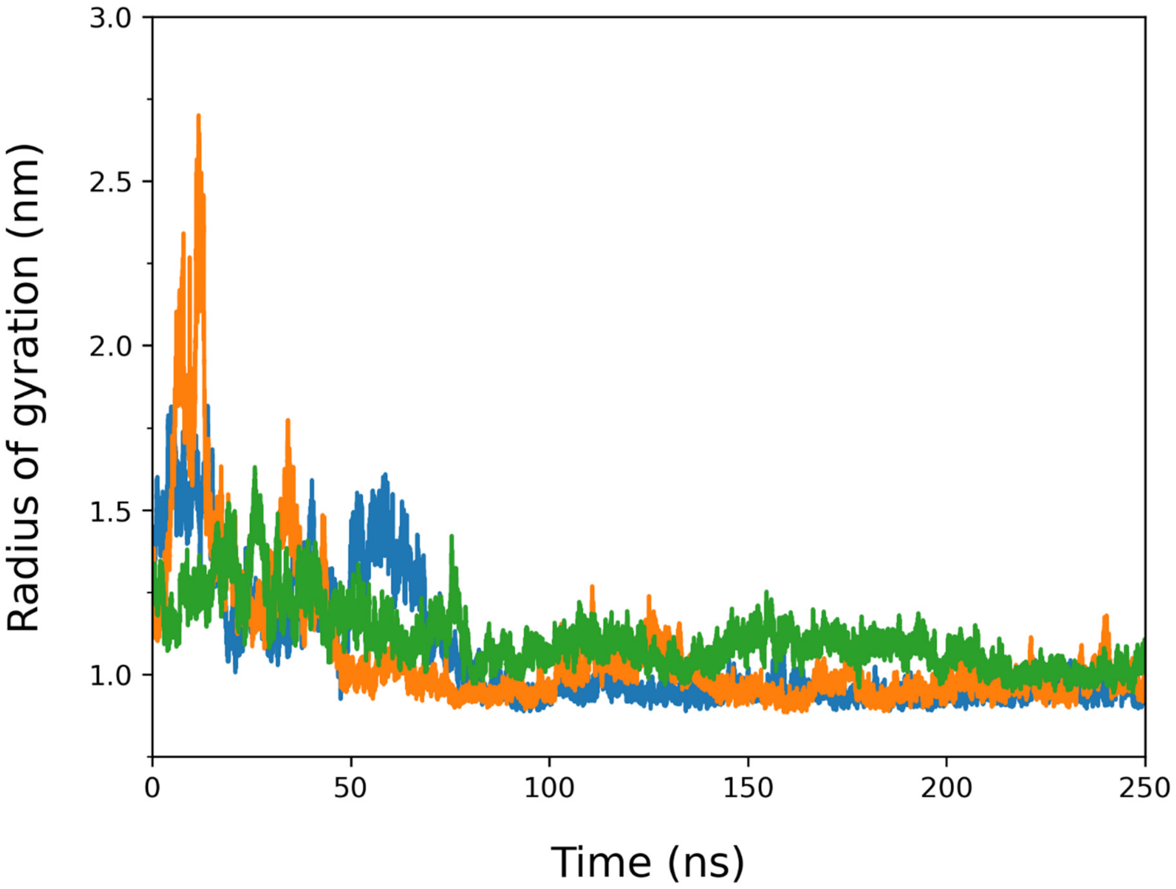

Supplementary Figure 6. MD of PA-DNA systems (see figure 6 of the main manuscript) The radius of gyration (Rg) was computed for the entire system across all three replicas. The blue curve represents the Rg for replica 1, the orange curve for replica 2, and the green curve for replica 3.

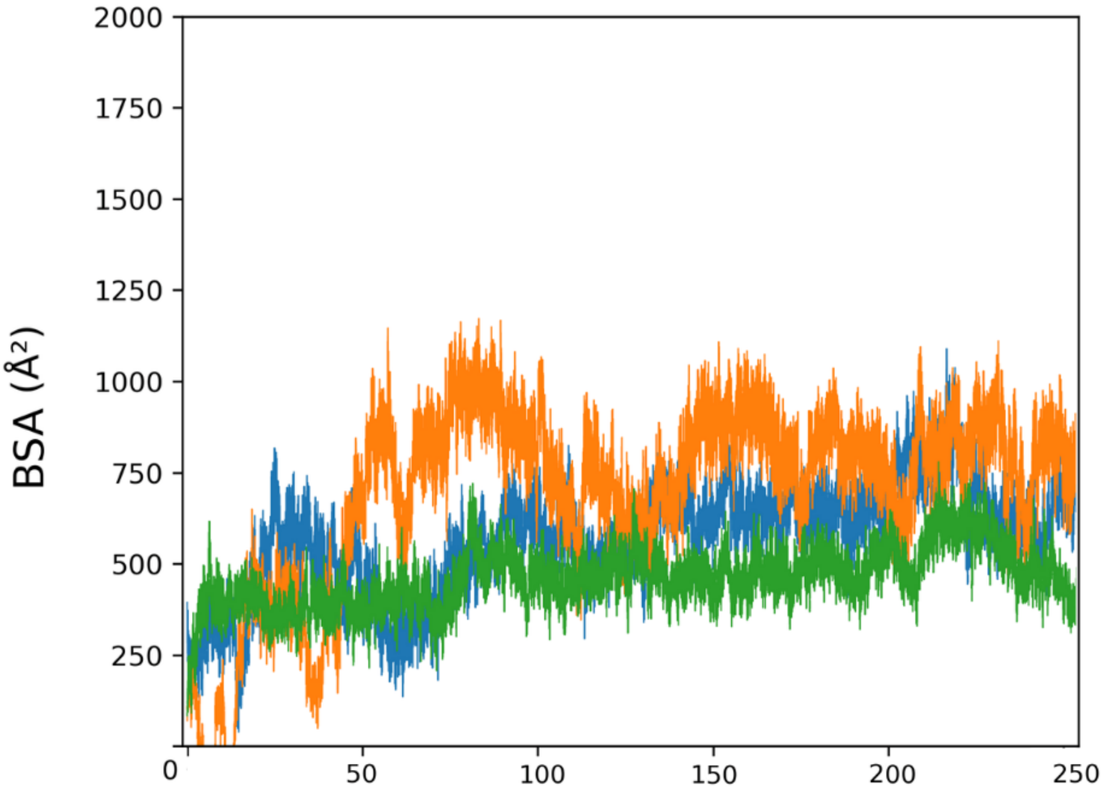

Supplementary Figure 7. The buried surface area (BSA) was computed for the systems across all three replicas of the MD of PA-DNA systems. The blue curve represents the BSA for replica 1, the orange curve for replica 2, and the green curve for replica 3.

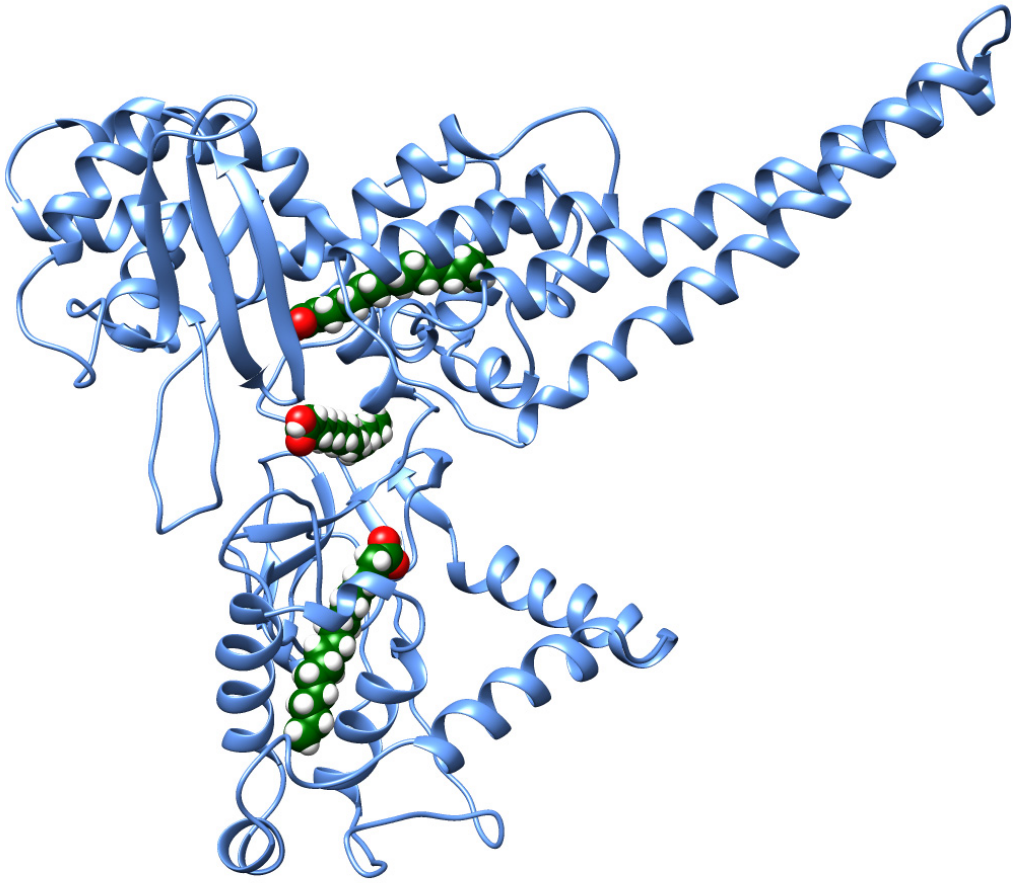

Supplementary Figure 8. A 3D representation of the three druggable sites of PA molecules (shown in Van der Waals representation) predicted by DynamicBind onto hTOP1 (shown in cyan cartoon). Note that the DNA cannot be included.

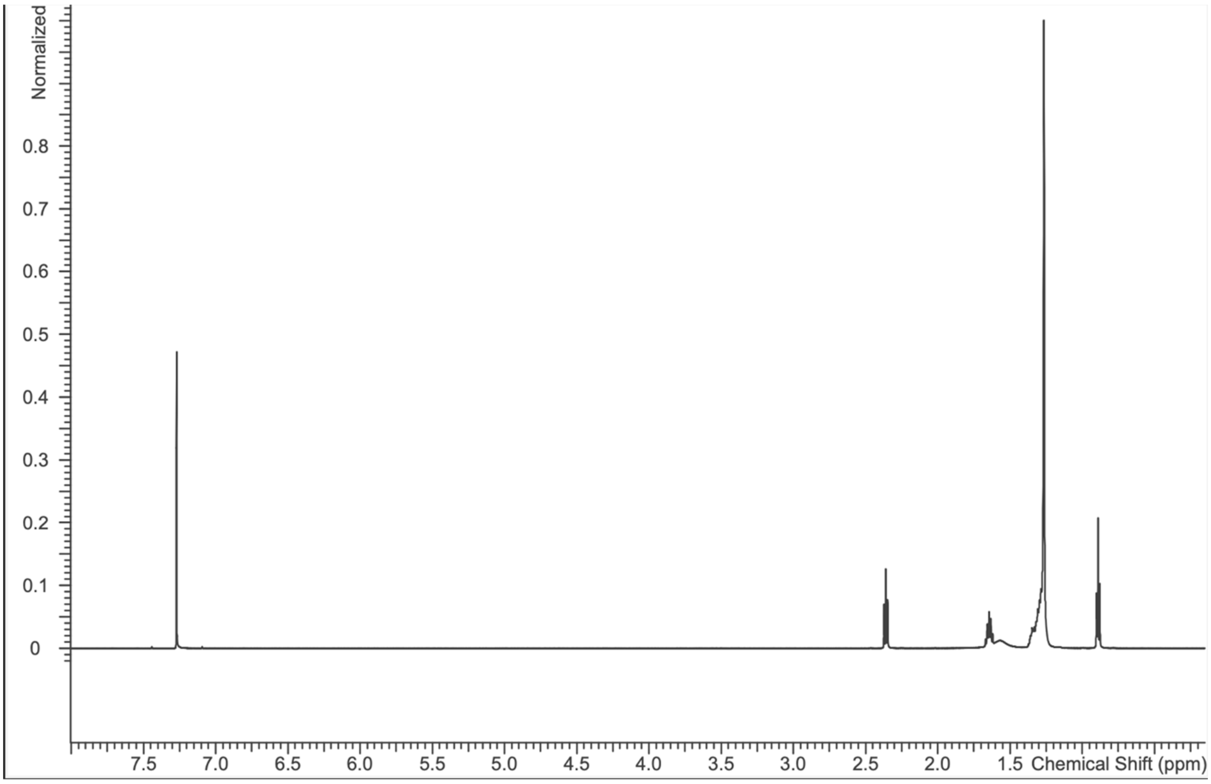

Supplementary Figure 9. ^{1}H- NMR spectrum of PA purification step.
